# Diffusion-Weighted Imaging Prior to Percutaneous Sclerotherapy of Venous Malformations—Proof of Concept Study for Prediction of Clinical Outcome

**DOI:** 10.3390/diagnostics12061430

**Published:** 2022-06-09

**Authors:** Mirjam Gerwing, Philipp Schindler, Kristian Nikolaus Schneider, Benedikt Sundermann, Michael Köhler, Anna-Christina Stamm, Vanessa Franziska Schmidt, Sybille Perkowski, Niklas Deventer, Walter L. Heindel, Moritz Wildgruber, Max Masthoff

**Affiliations:** 1 Clinic for Radiology, University of Muenster and University Hospital of Muenster, 48149 Muenster, Germany; philipp.schindler@ukmuenster.de (P.S.); benedikt.sundermann@uni-oldenburg.de (B.S.); michael.koehler@ukmuenster.de (M.K.); anna-christina.stamm@ukmuenster.de (A.-C.S.); walter.heindel@ukmuenster.de (W.L.H.); moritz.wildgruber@med.uni-muenchen.de (M.W.); max.masthoff@ukmuenster.de (M.M.); 2 Department of Orthopedics and Tumor Orthopedics, University of Muenster and University Hospital of Muenster, 48149 Muenster, Germany; kristian.schneider@ukmuenster.de (K.N.S.); niklas.deventer@ukmuenster.de (N.D.); 3 Evangelisches Krankenhaus Oldenburg, Medical Campus University of Oldenburg, 26122 Oldenburg, Germany; 4 Research Centre Neurosensory Science, Carl von Ossietzky University Oldenburg, 26129 Oldenburg, Germany; 5 Department of Radiology, University Hospital, LMU Munich, 80336 Munich, Germany; vanessa.schmidt@med.uni-muenchen.de; 6 Clinic for Pediatric and Neonatal Surgery, University of Muenster and University Hospital of Muenster, 48149 Muenster, Germany; sybille.perkowski@ukmuenster.de

**Keywords:** venous malformation, slow-flow vascular malformations, radiomics, percutaneous sclerotherapy, quality of life

## Abstract

Prediction of response to percutaneous sclerotherapy in patients with venous malformations (VM) is currently not possible with baseline clinical or imaging characteristics. This prospective single-center study aimed to predict treatment outcome of percutaneous sclerotherapy as measured by quality of life (QoL) by using radiomic analysis of diffusion-weighted (dw) magnetic resonance imaging (MRI) before and after first percutaneous sclerotherapy. In all patients (*n* = 16) pre-interventional (PRE-) and delta (DELTA-) radiomic features (RF) were extracted from dw-MRI before and after first percutaneous sclerotherapy with ethanol gel or polidocanol foam, while QoL was assessed using the Toronto Extremity Salvage Score (TESS) and the 36-Item Short Form Survey (SF-36) health questionnaire. For selecting features that allow differentiation of clinical response, a stepwise dimension reduction was performed. Logistic regression models were fitted and selected PRE-/DELTA-RF were tested for their predictive value. QoL improved significantly after percutaneous sclerotherapy. While no common baseline patient characteristics were able to predict response to percutaneous sclerotherapy, the radiomics signature of VMs (independent PRE/DELTA-RF) revealed high potential for the prediction of clinical response after percutaneous sclerotherapy. This proof-of-concept study provides first evidence on the potential predictive value of (delta) radiomic analysis from diffusion-weighted MRI for Quality-of-Life outcome after percutaneous sclerotherapy in patients with venous malformations.

## 1. Introduction

Venous malformations (VM) are slow-flow vascular malformations according to the ISSVA (International Society for the Study of Vascular Anomalies) classification, with a prevalence of up to 1% in the overall population [1,2,3]. VM are congenital lesions consisting of dysplastic and dilated veins due to a disturbed vasculogenesis. If symptomatic, common symptoms include pain, swelling, inflammation and functional impairment. Since vascular malformations typically involve multiple tissue compartments such as subcutaneous fat, muscles or even bone, surgical resection is challenging and frequently associated with recurrence of the lesion. In recent years, percutaneous sclerotherapy of VMs has emerged as a minimally invasive treatment option, using sclerosing agents such as (gelified) ethanol, polidocanol or bleomycin [4]. These agents harm dysplastic endothelial cells of VMs, causing intralesional thrombosis, inflammation, and scarring. Percutaneous sclerotherapy has proven to be effective even in challenging anatomic regions accompanied by a low risk profile [2]. However, many patients require several sessions of percutaneous sclerotherapy before improvement of clinical symptoms may be recognized. Moreover, some patients do not sufficiently respond to percutaneous sclerotherapy, with substantial implications for patients’ daily and social lives. 

Therefore, early identification of responders and non-responders to percutaneous sclerotherapy is crucial. Currently, no reliable baseline clinical or imaging parameters exist to predict patient outcome following percutaneous sclerotherapy. Imaging in VMs is based on ultrasound and magnetic resonance imaging (MRI) [5]. Since diffusion-weighted (dw) magnetic resonance imaging (MRI) depends on the intralesional diffusion of water molecules, which may be restricted in slow-flow malformations either by partial thrombosis or after sclerotherapy, dw-MRI may provide useful predictive parameters for the effectiveness of sclerotherapy [6,7]. In this context, data on radiomics approaches for the assessment of the predictive value of MRI are still sparse and usually, the radiomics method uses pre-therapeutic images for evaluation of prediction, which neglects the changes of the observed disease during treatment or follow-up [8]. Hence, an additional delta-radiomics approach, which employs the changes in radiomic features during treatment to instruct clinical decisions, may be worthwhile for evaluation of treatment response [8,9]. To the best of our knowledge, there is no data on the capability of baseline and delta-radiomic features of dw-MRI to predict the treatment response of percutaneous sclerotherapy in VM patients.

Therefore, this study aims to evaluate the predictive value of radiomic features obtained from pre- and post-interventional dw-MRI in evaluating outcome of percutaneous sclerotherapy of VM by means of patients’ quality-of-life improvement. 

## 2. Materials and Methods

### 2.1. Study Design

**Figure 1:**
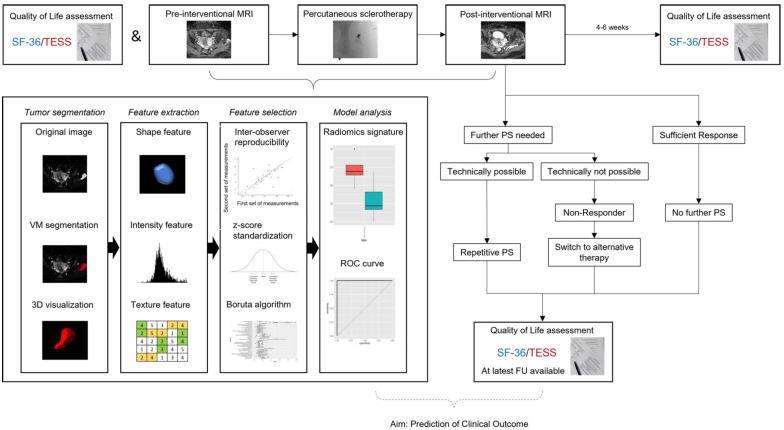
Scheme on study workflow. Percutaneous sclerotherapy was repeated if patients suffered from remaining symptoms and treatable areas of the VM were identified on ultrasound imaging. SF-36: Short Form-36, TESS: Toronto Extremity Salvage Score in Unoperated Controls, PS: Percutaneous Sclerotherapy, FU: Follow-Up, VM: Venous Malformation, ROC: Receiver Operating Characteristic. Modified to Yang et al. [10].

This study was performed as a prospective single-center observational proof-of-concept study at a tertiary care vascular anomalies center. All patients with venous malformations of the upper or lower limb who had never been treated by percutaneous sclerotherapy before and who were not treated otherwise for the past 12 months were consecutively included between March 2020 and June 2021. Patients with mix-type vascular malformations were excluded. Children too young for MRI without sedation or anesthesia were also excluded. Diagnosis of VM was based on clinical and imaging (ultrasound, MRI) assessment. Percutaneous sclerotherapy was performed in all patients for clinical indications after interdisciplinary vascular anomalies board discussion independent of the presented study. Patient data and procedural data were retrieved from the Clinical and Radiological Information System (CIS, RIS) and Picture Archiving and Communication System (PACS). The study was approved by the Institutional Review Board (ID: 2019-515-f-S). Figure 1 shows the study workflow. 

### 2.2. MRI Examination

MRI was performed within 48 h before and 24 h after first percutaneous sclerotherapy of the VM. All imaging examinations were conducted on a 1.5 T scanner (Ingenia, Philipps, Best, The Netherlands) with an anterior coil and a build-in posterior coil (32 channels) covering the entire VM (see Table 1 for anatomical locations). An axial T2 Dixon turbo spin echo (TSE) sequence was acquired for anatomical information, as well as short-TI inversion recovery (STIR) for fat suppression. Diffusion weighted images were acquired as axial diffusion-weighted whole-body imaging with background body signal suppression (DWIBS) using three b-values (0, 50, 1000; TE 71 ms, TR 4690 ms, IR 180 ms, EPI single shot, EPI factor 31, slice thickness 6 mm, slice gap 0 mm, ACQ voxel MPS 3.98/4.38/6.00 mm, REC voxel MPS 2.19/2.19/6.00 mm, sense factor 2). The calculated apparent diffusion coefficient map was used for further analysis. The examination was conducted without contrast medium. The same protocol was used for MRI before and after percutaneous sclerotherapy.

### 2.3. Interventional Therapy

All procedures were performed after informed consent. Patients received analgosedation (midazolam, piritramide) or general anesthesia. For percutaneous sclerotherapy, one to five appropriate locations of VMs were sequentially identified under ultrasound-guidance. Intravascular placement of the inserted 22 G needle was assured with aspiration of venous blood and injection of contrast medium under fluoroscopy, confirming appropriate distribution within the venous malformation and excluding extravasation. Sclerotherapy was performed with gelified ethanol (DiscoGel, Ab Medica Deutschland GmbH & Co. KG, Düsseldorf, Germany; containing gelified 96% ethyl alcohol) and/or polidocanol foam (Aethoxysklerol 3%; Kreussler Pharma, Wiesbaden, Germany). Percutaneous sclerotherapy was repeated after 4 to 6 weeks if it was technically feasible, patients reported restricting symptoms, mostly pain, or no further improvement in patients’ symptoms was gained after sclerotherapy. 

All procedural data and patient recordings of performed interventional procedures were analyzed with regard to age, sex, localization (upper/lower limb), number of used accesses (needle locations) during sclerotherapy, type and amount of sclerosing agent, as well as any minor or major adverse events according to the Common Terminology Criteria for Adverse Events, version 5.0 classification.

### 2.4. Clinical Outcome and Quality-of-Life

Clinical outcome and quality-of-life were assessed using two standardized questionnaires (SF-36 and TESS score) at baseline, four weeks after the first interventional treatment of the venous malformation and at latest available follow-up (range 5–20 months, information on the latest follow up can be found in Table 1). The 36-Item Short Form Survey (SF-36) health questionnaire is a 36-item generic measure of general health status [11]. It covers physical and mental measures and is used for a wide range of different evaluations, including relative burden of a disease or assessment of therapy outcome [12,13]. For patients under 18 years of age, the questionnaires were completed with the aid of their parents. The Toronto Extremity Salvage Score (TESS) is widely used for the functional assessment of patients following surgery for musculoskeletal tumors located at the upper or lower extremity [14,15,16]. It comprises questions regarding mostly everyday life tasks and evaluates the difficulty in performing these tasks. Additional questions cover challenges in completing duties at work and leisure activities. 

The quality-of-life data analysts were blinded for imaging and interventional procedure data. With pain and limitations in motility being the major symptoms of VM located at the extremities, affiliated parameters from quality-of-life data were used to define, in total, four outcome parameters to assess: (1) effectiveness of first percutaneous sclerotherapy and (2) effectiveness of repetitive percutaneous sclerotherapy. Effectiveness of the first percutaneous sclerotherapy was defined by two outcome parameters: first, improvement in SF-36 pain score by at least 20 points and second, improvement in TESS score by at least 5% each four weeks after first sclerotherapy compared to baseline. Effectiveness of repetitive percutaneous sclerotherapy was defined by two outcome parameters: first, achieving a SF-36 pain score of at least 85 points (meaning pain only rarely or in severe stress situations) and second, by achieving a TESS score of 85% or higher. 

### 2.5. Image Analysis and Radiomic Feature Extraction

Two experienced radiologists, blinded for interventional and clinical data, independently evaluated pre- and post-interventional MRI data in a random order. For image segmentation, the reader-specific label map volume based on the VM volume in T2-weighted images was transferred to diffusion-weighted-MRI sequences. Radiomic features from labelled dw-MRI sequences (ADC) were extracted twice, each by the same independent readers for inter-observer analysis, and included 162 first-order logic features and 216 gray level co-occurrence matrix (GLCM) features, as described elsewhere [17]. The radiomics features were extracted from the VM regions on both pre- (PRE-RF) and post-interventional (POST-RF) dw-MRI. The delta-radiomic features (DELTA-RF) were calculated by subtracting PRE-RF from POST-RF. Image analysis and feature extraction was performed with a freely available software package (3D slicer, version 4.11.2, USA [18])

### 2.6. Radiomic Feature Selection and Dimension Reduction for Differentiation of Response to Interventional Therapy

Due to the exceeding number of radiomic features (*n* = 378) in comparison to the number of patients (*n* = 16), feature selection and dimension reduction were performed separately for PRE-RF and DELTA-RF [19]. Inter-observer reproducibility of the textural features was assessed by calculating the Concordance Correlation Coefficient (CCC) for each of the features as a measure of the intra-class correlation. Features with a coefficient ranging from 0.8 to 1 were considered “excellent” and included in further analysis [20,21]. A z-score standardization was used for feature normalization, followed by a random subdivision of the dataset in a balanced training and test dataset (75/25 ratio). Further feature reduction was performed only on the training dataset using a Boruta machine-learning algorithm, which applies a Random Forest algorithm by performing a top-down search for relevant features. Irrelevant features are progressively eliminated to stabilize the model after comparison of original attributes’ importance to importance achievable at random [22]. A correlation matrix was calculated subsequently and closely correlated features were eliminated [23,24]. In a last step, features that allow for differentiation of clinical response according to the four defined outcome parameters from quality-of-life analysis (see above) were selected respectively and logistic regression models were fitted. Diagnostic accuracy of the features was evaluated by receiver operating characteristic (ROC) calculating the area-under-the-curve (AUC). Radiomic feature selection and dimension reduction was performed by using an open-source software package (R/R studio, version 4.0.5; R Foundation, Vienna, Austria).

### 2.7. Statistical Analysis

All data are presented as mean ± standard deviation or as median (and range), as appropriate. The paired student *t*-test was used for pre- versus postinterventional data. The characteristics age, sex, localization of the malformation (upper or lower limb), previous therapies, number of accesses during sclerotherapy, sclerosing agent (gelified ethanol, polidocanol or a combination of both), the quantity of the agent in mL, and the number of performed therapies were analyzed regarding their influence on patient outcome (improvement in SF-36 pain score by at least 20 points or improvement in TESS score by at least 5% each four weeks after first sclerotherapy compared to baseline; achievement of a SF-36 pain score of at least 85 points; or a TESS score of 85% or higher after repetitive sclerotherapy) with a binary logistic regression using IBM SPSS statistics (version 28.0.1.0, IBM Corp., Armonk, NY, USA) for metric and categorial predictors, respectively. Statistical analysis of the QoL data was performed using Prism 9 software (GraphPad Software Inc., San Diego, CA, USA). A *p*-value of < 0.05 was considered significant. 

## 3. Results

### 3.1. Patient Characteristics

**Figure 2:**
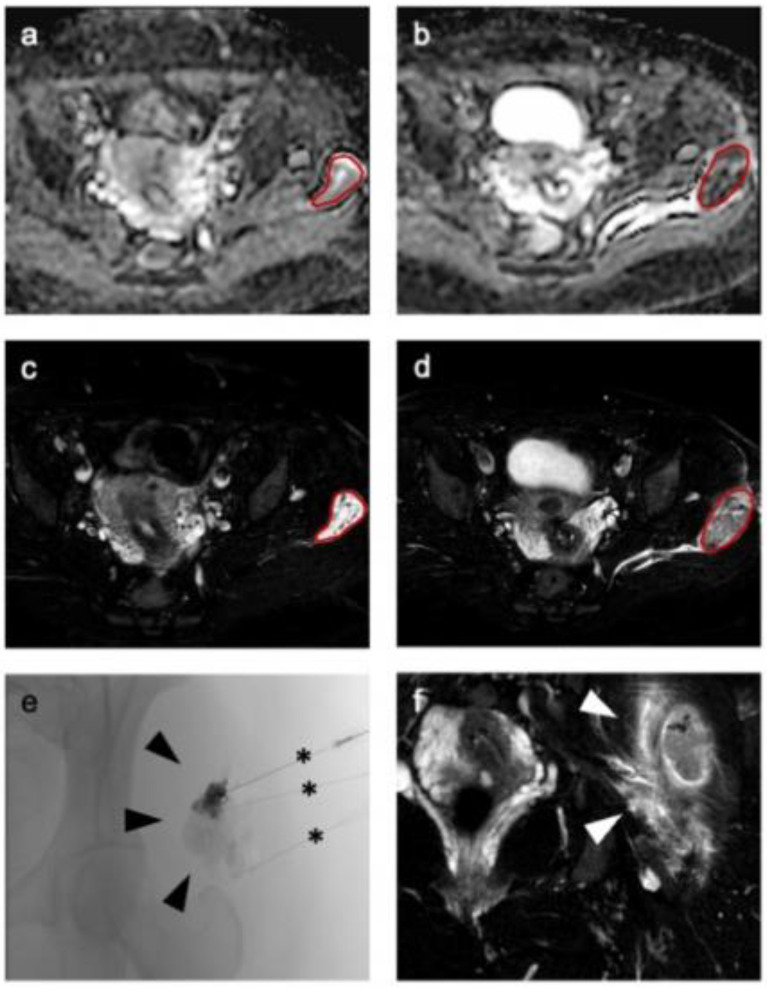
Representative imaging of a venous malformation in the gluteal area of a 34-year-old woman before therapy (**a**) shows a well marginated lesion on apparent diffusion coefficient (ADC) (**a**) and T2-weighted (**c**) imaging, which is markedly hypo-intense after therapy in ADC (**b**) and T2-weighted imaging (**d**,**f**) with a surrounding hyperintense edema (arrowheads in (**f**)). For segmentation a volume of interest (red line) was drawn on T2-weighted images (**c**,**d**) and transferred to ADC (**a**,**b**). (**e**) Representative fluoroscopic imaging during percutaneous sclerotherapy followed by administration of the sclerosing agent via percutaneous needles in three locations (black asterisks).

Sixteen consecutive patients (12 female and 4 male; median age 27 years [range 8–49 years]) undergoing percutaneous sclerotherapy for venous malformation were included in this study. Nine patients (56.25%) had a VM of the upper extremity, seven patients (43.75%) of the lower extremity. Three patients had a history of previous treatment at least 9 or more years ago (patients 6 and 15 had two surgical treatments previously, patient 6 in the 1970s and patient 15 in 2004, patient 14 one surgical treatment (2011) and one laser therapy (2012)). Only one patient received additional laser therapy after sclerotherapy (patient 2); all other patients had no further treatment. Median follow up was 12 months after the first treatment, range 5–20 months. Last quality-of-life assessment was at least 4 weeks after last treatment. Detailed information on patient characteristics and follow-up can be found in Table 1 summarizing both baseline patient data as well as information on performed procedures. Additionally, exemplary MRI and procedural images of a patient with a venous malformation treated by sclerotherapy is presented in Figure 2. 

### 3.2. Interventional Therapy

A mean of 2.6 ± 1.1 (range 1–4) percutaneous sclerotherapies were performed per patient. Patients received sclerotherapy in a mean of 2.7 ± 1.4 locations per session, range 1–5. In the first therapy cycle, six patients received only gelified ethanol with a dose of 1.9 ± 1.1 mL, while seven patients were treated with polidocanol alone, with a dose of 2.9 ± 1.1 mL. The remaining three patients were treated with a combination of 2.3 ± 0.6 mL gelified ethanol and 1.7 ± 0.6 mL polidocanol. No major adverse events were observed during or after the intervention, and minor adverse events included post-interventional pain and local inflammation. No procedure-associated short- or long-term adverse events, such as necrosis or nerve damage, were observed during follow-up. 

### 3.3. Clinical Outcome—Quality-of-Life

**Figure 3:**
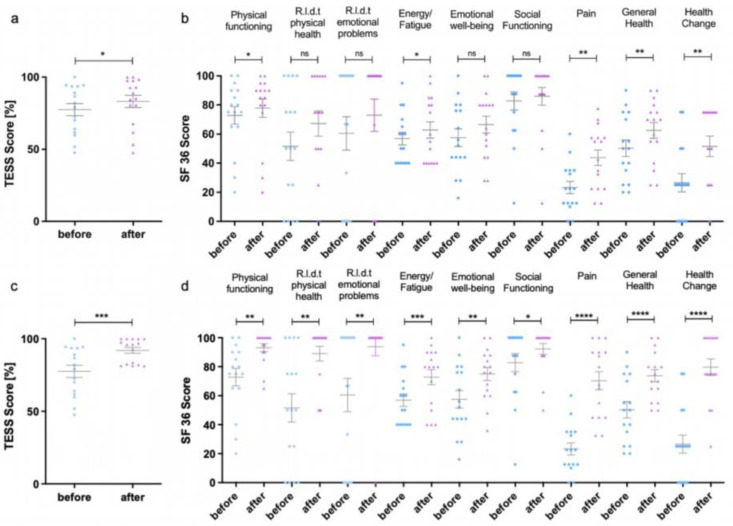
Results of the Toronto Extremity Salvage Score (TESS) (**a**,**c**) and Short Form 36 (SF-36) (**b**). (**d**) survey after the first percutaneous sclerotherapy (**a**,**b**) and after repeated therapies (**c**,**d**). The individual values can be found in Supplementary Materials Table S1. * *p* < 0.05, ** *p* < 0.01, *** *p* < 0.001, **** *p* < 0.0001 R.l.d.t. = Role limitations due to.

Detailed quality-of-life data at baseline, four weeks after first sclerotherapy and at last follow-up at least four to six weeks after the last sclerotherapy, can be found in Figure 3. The detailed values for the assessed categories, as well as statistical details, can be found in Supplementary Materials Tables S1 and S2. TESS showed a significant improvement in quality-of-life at last follow-up after repetitive sclerotherapy (92.0 ± 8.1%) in comparison to the baseline (77.5 ± 16.2%, *p*-value 0.0009). Thus, the improvement led to score values close to the maximum of 100%. The more-detailed SF-36 survey achieved a significant increase in the score values in all assessed categories after repetitive sclerotherapy as well. In detail, the score for physical functioning significantly improved from 72.8 ± 23.3 to 93.1 ± 11.1 (*p*-value 0.0022), while the score for the role limitations due to physical health increased significantly from 51.6 ± 38 to 89.1 ± 20.4 (*p*-value 0.0012). Three patients did not sufficiently improve in these categories, but already had rather high baseline scores for physical functioning of 75 or more. With the SF-36 score, the effects of physical disease on mental health are also evaluated, as characterized by the following categories: role limitations due to emotional problems, energy/fatigue, emotional well-being and social functioning. All four categories improved significantly after repetitive sclerotherapy, from 60.4 ± 45.9 to 93.8 ± 25 (*p*-value 0.0083), from 56.9 ± 17.5 to 72.8 ± 20.9 (*p*-value 0.0006), from 57.5 ± 24.1 to 75.0 ± 18.0 (*p*-value 0.0024), and from 82.8 ± 25.0 to 92.2 ± 15.1 (*p*-value 0.0346), respectively. Additionally, repetitive percutaneous sclerotherapy led to a significant improvement of pain, yielding an increase in SF-36 score from 23.3 ± 16.7 to 70.3 ± 24.8 (*p*-value < 0.0001). Here, half of the patients had a score of 80 or higher at final follow-up, which means pain occurs only in situations with increased stress, such as extensive participation in sports or long walks. As an overall score, the general health score improved significantly from 50.3 ± 22.6 to 73.8 ± 16.8 (*p*-value < 0.0001). Furthermore, patients reported a considerably more positive outlook regarding their expectations for future health change with a score increasing from 26.6 ± 25.0 at baseline to 79.7 ± 22.8 after repetitive sclerotherapy (*p*-value < 0.0001). In summary, defined quality-of-life outcome parameters were achieved as follows: eight patients (50%) improved in pain score by at least 20 points and six patients (37.5%) showed an improvement in TESS score of at least 5% after first sclerotherapy. Meanwhile, seven patients (43.75%) achieved a SF-36 pain score of 85 or higher, and 10 patients (62.5%) achieved a TESS score of 85% or higher at last follow up after repetitive sclerotherapy. All other patients were defined as non-responders for further analysis.

### 3.4. Outcome Prediction–Binary Logistic Regression

Binary logistic regression identified none of the analyzed common clinical parameters such as age, sex, location of VM, prior therapies, number of accesses during sclerotherapy, type or amount of sclerotic agent used, or number of performed sclerotherapies as predictive for any of the above defined quality-of-life outcome measures (all *p*-values > 0.05, see Supplementary Materials Table S2). Hence, there are no common baseline patient characteristics that can be used to predict response to percutaneous sclerotherapy of VM. Therefore, radiomic analysis of the data was performed subsequently (an overview of the workflow can be found in Figure 1).

### 3.5. Outcome Prediction—Radiomics Features before Therapy

**Figure 4:**
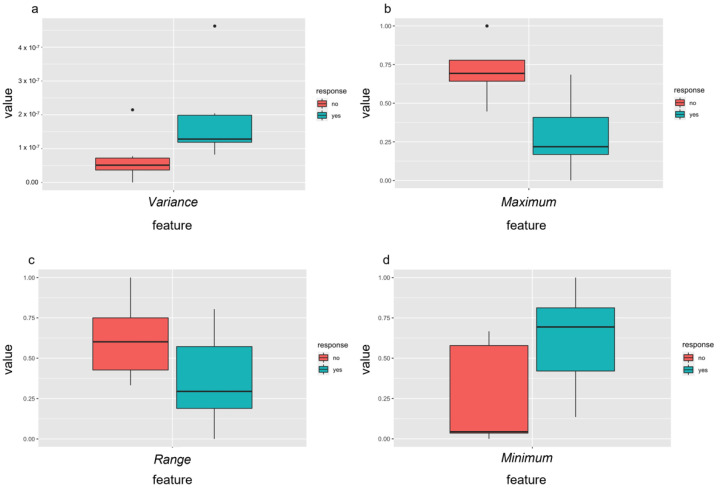
Independent radiomic features of pre-treatment MRI differentiated well between responders (yes = green) and non-responders (no = red) to percutaneous sclerotherapy using the identified features after feature selection. Response was defined as an increase in pain score of at least 20 points assessed with the SF-36 (**a**) or improvement of the TESS score of at least 5% (**b**) after the first treatment and as an achievement of at least 85 points in the SF-36 pain score (**c**) or of 85% or more in the TESS score (**d**) after repetitive percutaneous sclerotherapy.

Radiomic features are written in italics for easy identification. In a first step, radiomics analysis of baseline dw-MRI data (PRE-RF) was performed to predict outcome after sclerotherapy of VM. Subsequent to the multistep dimension reduction from pre-interventional dw-MRI data only, independent PRE-RF related to each of the defined outcome goals were identified (Figure 4). Regarding response to the first percutaneous sclerotherapy (PRE-RF), the feature *variance* was identified for an increase of at least 20 points in SF-36 pain score and the feature *maximum* was identified for an increase in TESS score of at least 5% (Figure 4). Regarding response at last follow-up after repetitive percutaneous sclerotherapy, the feature *range* was identified for an achieved SF-36 pain score of at least 85 (meaning no significant pain in everyday life) and the feature *minimum* was identified for an achieved TESS score of at least 85% (Figure 4). When assessing the prediction performance of the aforementioned tissue factors, each ROC analysis for the discrimination of responders and non-responders to percutaneous sclerotherapy according to the defined outcome parameters showed an accuracy with an AUC of 1, respectively.

### 3.6. Outcome Prediction—Delta Radiomic Features before and after the First Treatment

**Figure 5:**
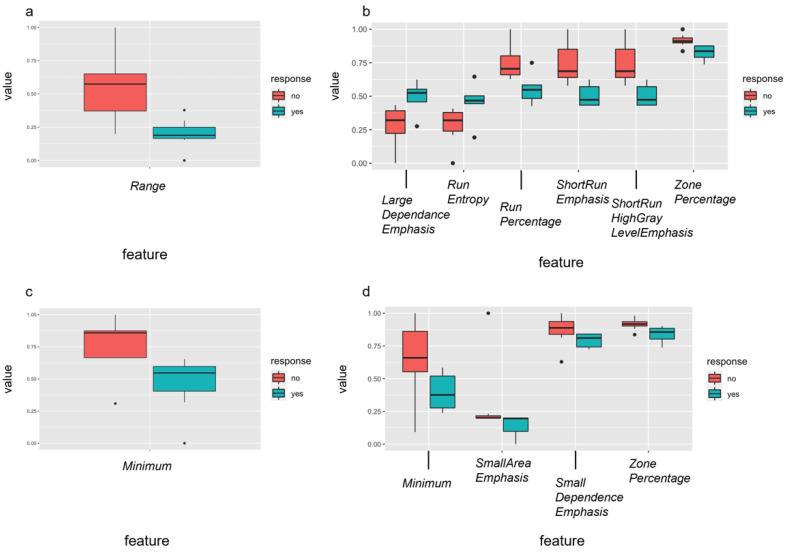
Independent delta-radiomic features of pre-treatment MRI differentiated well between responders (yes = green) and non-responders (no = red) to percutaneous sclerotherapy using the identified features after feature selection. Response was defined as an increase in pain score of at least 20 points assessed with the SF-36 (**a**) or improvement of the TESS score of at least 5% (**b**) after the first treatment and as an achievement of at least 85 points in the SF-36 pain score (**c**) or of 85% or more in the TESS score (**d**) after repetitive percutaneous sclerotherapy. In case of several relevant features per response criterion (**b**,**d**), a correlation matrix was calculated (details can be found in Supplementary Materials Figure S1).

Radiomic features are written in italics for easy identification. In a next step, delta radiomics analysis from dw-MRI data before and after the first percutaneous sclerotherapy of VM was performed to predict outcome after sclerotherapy, obtaining DELTA-RF after the multistep dimension reduction (Figure 5). Regarding response to the first percutaneous sclerotherapy (DELTA-RF), the feature *range* was identified, discriminating responders from non-responders regarding an increase in SF-36 pain score by at least 20 points. Further, for an increase in TESS score after first sclerotherapy by at least 5%, six independent features (*large dependence emphasis, run entropy, run percentage, short run emphasis, short run high gray level emphasis, and zone percentage*) were identified (Figure 5). As clusters of DELTA-RF became apparent in the correlation matrix (Supplementary Figure S1), representing closely correlating features, only the combination of *short run emphasis* and *zone percentage* were included in further analysis. Regarding response at last follow-up after repetitive percutaneous sclerotherapy, four independent DELTA-RF (*minimum, small area emphasis, small dependence emphasis and zone percentage*) were identified with respect to achieving a SF-36 pain score of 85 or higher. Again, the correlation matrix revealed closely correlating features (Supplementary Materials, Figure S1), whereby the combination of *minimum, zone percentage* and *small area emphasis* were included for predictive performance analysis. For achieving a TESS score of at least 85%, the DELTA-RF *minimum* was identified, which is the same feature identified for the PRE-RF analysis (see above). When assessing the predictive value of these DELTA-RF, the ROC analysis for the discrimination of responders and non-responders to the chosen response criterion each revealed an accuracy with an AUC of 1, respectively.

## 4. Discussion

Although venous malformations (VM) are benign lesions, they may cause severe symptoms such as pain, swelling, or functional impairment once symptomatic. As congenital lesions with a high rate of recurrence after therapy or the impossibility of definitive cure, VM often accompany patients’ lives over a tedious period with alternating or chronic symptoms [25]. Thus, patients often experience a high degree of physiological and psychological strain, aggravated by a high rate of initial misdiagnosis or insufficient therapies [26]. This notion is verified by the poor baseline quality-of-life data (especially regarding pain or psychological scores) of the patient cohort presented in this study, showing values close to the ones reported for severe bone lesions or musculoskeletal cancers [27,28]. However, different from standardized response assessment in musculoskeletal cancers, response assessment early after interventional treatment of VMs is a challenge, as therapy success is often not visible on imaging. In this study, a thorough analysis of patients’ quality-of-life by well-established SF-36 and TESS surveys was performed to assess response to percutaneous sclerotherapy and generate meaningful targets for outcome prediction.

Percutaneous sclerotherapy has been established as a minimally invasive strategy with good outcomes and a low risk profile [4], fostering it as treatment of choice in most VMs, even in challenging locations [2]. This notion is supported by the good outcome results in the presented study, as shown by significant improvement in patients’ quality-of-life. However, there are currently no established baseline patient characteristics or imaging parameters that may predict such responses prior to VM treatment [29]. Similarly, all common baseline parameters, as well as interventional procedure data, were not associated with patient outcome neither after first sclerotherapy nor at last available follow-up after repetitive cycles of sclerotherapy in the presented study. However, predictive biomarkers to identify non-responders prior to or early during the repetitive cycles of sclerotherapy would be crucial to guide treatment decisions in VM patients and thereby protect patients from (further) inefficient treatment sessions.

Therefore, this study evaluated the feasibility of using quantitative tissue parameters extracted from pre- (PRE-RF) and post-interventional (DELTA-RF) dw-MRI for response prediction to percutaneous sclerotherapy, as diffusion-weighted (dw) imaging with calculation of the apparent diffusion coefficient is a sensitive MRI method to detect venous malformations [30]. Dw-MRI is influenced by a variety of factors, such as cell density, intralesional thrombosis [31] or perfusion [32] and, as shown from data of this study, by sclerosing agents used for percutaneous sclerotherapy. Further, visual assessment of dw-MRI carries pitfalls such as influence by factors like T2 relaxation times [33], so a radiomics approach enables a more objective and thorough multidimensional assessment of these data. Artificial intelligence has already been used for vascular diseases (peripheral artery disease or deep vein thrombosis of the limb [34].

Using radiomics analysis of baseline dw-MRI as well as change in dw-MRI data after sclerotherapy, this study identified several (pre- or delta-) radiomic features significantly associated with treatment response (as characterized by the defined outcome parameters in quality-of-life improvement) after either first sclerotherapy or repetitive cycles of sclerotherapy. In detail, identified pre-interventional features (*range, minimum, maximum*) indicate that the heterogeneity of the untreated VMs in the baseline dw-MRI scan is predicting outcome of sclerotherapy. This notion fits well with the clinically observed heterogeneity of these lesions. Hence, once confirmed in larger prospective studies, these features may guide therapeutic decision for or against starting a treatment with percutaneous sclerotherapy.

Further, delta-radiomics analysis identified several features associated again with changes in heterogeneity (*minimum, range*), thereby results are in line with observations for baseline radiomics analysis, and more advanced features of heterogeneity such as change in *zone percentage* were identified as associated with outcome response of percutaneous sclerotherapy. Exemplarily, this feature was also found to be relevant in response prediction to neo-adjuvant chemoradiation of lymph node metastases in non-small cell lung cancer (NSCLC) [35]. Hence, delta radiomics analysis of dw-MRI data before and after first sclerotherapy also proved feasible in outcome prediction. The identified features, once confirmed in larger prospective studies, may guide therapeutic decision to continue sclerotherapy or change treatment to other available strategies.

Both baseline and delta radiomics analysis emphasize the potential of dw-MRI to guide treatment decision and therapy monitoring via assessing imaging biomarkers beyond the common anatomical information and including the advantages of a non-invasive diagnostic tool without the need for radiation or contrast agents, which is of benefit considering the usually young patient cohort with the need for repetitive imaging [36]. Thereby, the technique shows comparable potential to recently introduced multispectral optoacoustic imaging (MSOT) for VM therapy monitoring, but with dw-MRI being currently much more available and performable on every standard MRI scanner [37].

The presented study has several limitations. First, it is limited by a small patient cohort in a single vascular anomalies center, resulting in a small number of patients for the training and test set of the performed radiomics analysis. Although a multistep dimension reduction approach was only performed in the training dataset to ensure the generalizability of the statistical model, the number of patients in the test dataset is small. The AUC of 1 achieved in the prediction performance analysis is therefore due to the small test cohort and is not intended to suggest that a perfect model has been developed here. However, considering the rarity of disease, inclusion of only untreated and symptomatic patients with VMs of the limbs old enough to perform MRI on the identical scanner before and after sclerotherapy, without the need for sedation or anesthesia, to avoid any bias from various pretreatments, different scanner hardware or imaging regions or the need of repetitive anesthesia, the number of included patients is consistent. Thereby, the study provides proof-of-concept data to guide future larger prospective multicenter studies by identifying dw-MRI as potential predictive tool for treatment response in VM patients undergoing sclerotherapy.

Second, two different sclerotic agents were used for sclerotherapy, which, however, is frequently performed in percutaneous sclerotherapy therapy to take advantage of certain properties of the respective agents [2,4]. Importantly, in perspective, dw-MRI could also be evaluated for monitoring other treatment options of vascular anomalies.

## 5. Conclusions

In conclusion, the presented study shows, as proof-of-concept, that diffusion-weighted MRI has the potential to predict therapy response after percutaneous sclerotherapy of venous malformations. Once confirmed in larger studies, the identified radiomic features may help to guide therapeutic decisions based on the most important outcome—the improvement of patients’ quality-of-life.

## Figures and Tables

**Table 1:** Patient characteristics. y = years, F = female, M = male, No = number, mo = months.

ID	Age, y	Sex	Localization	Previous Therapy	No of Used Accesses	Sclerosing Agent	Quantity, mL	No of Performed Therapies	Follow-up, mo
1	22	F	forearm	none	4	gelified ethanol/polidocanol	2/2	2	19
2	24	F	lower leg	none	1	gelified ethanol	1	1	17
3	9	M	elbow	none	2	polidocanol	2	2	18
4	8	F	lower leg	none	3	polidocanol	2	2	17
5	46	F	knee	none	4	gelified ethanol/polidocanol	3/1	4	20
6	48	M	forearm	resection	3	polidocanol	2	3	16
7	25	F	thigh	none	1	gelified ethanol	2	4	15
8	17	F	forearm	none	2	gelified ethanol	2	1	12
9	21	F	forearm	none	1	gelified ethanol	2	1	9
10	25	F	forearm	none	1	gelified ethanol	1	3	12
11	26	F	thigh	none	4	gelified ethanol	4	2	10
12	34	F	thigh	none	3	polidocanol	4	3	8
13	15	M	thigh/knee	none	5	polidocanol	4	4	12
14	39	F	forearm	resection/laser therapy	1	polidocanol	4	3	5
15	18	F	thoracic wall	resection	4	gelified ethanol/polidocanol	2/2	3	8
16	49	M	forearm	none	4	polidocanol	4	4	5

## Data Availability

The raw data presented in this study are available on reasonable request from the corresponding author.

## Supplementary Materials

The following supporting information can be downloaded at: https://www.mdpi.com/article/10.3390/diagnostics12061430/s1, Figure S1: In case of several relevant features per response criterion a correlation matrix was calculated for achievement of an increase in TESS score of at least 5% after the first therapy (DELTA-RF) (a) or achievement of a pain score of at least 85 points assessed with the SF-36 (b). Correlogram including independent radiomic features where clusters of textural features became apparent. These indicate a strong correlation between parameters of the same imaging method. Blue circles indicate positive correlation, red circles negative correlation. Table S1: Statistical data of the QoL surveys after the first percutaneous sclerotherapy (a) and after repetitive percutaneous sclerotherapy (b). Table S2: Outcome details of the individual patients (1—outcome was achieved, 0—outcome was not achieved) after the first percutaneous sclerotherapy (improvement in SF-36 pain of at least 20 points or improvement in TESS score of at least 5%) and after repetitive percutaneous sclerotherapy (achievement of a pain score of at least 85 points assessed with the SF-36 or of a TESS score of at least 85%). Table S3: Outcome prediction with binary logistic regression revealed no statistical significance regarding basic characteristics of patients or the interventional procedure regarding outcome after the first percutaneous sclerotherapy (a) or after repetitive percutaneous sclerotherapy (b).
