# Supplementary material for: Diffusion-Weighted Imaging Prior to Percutaneous Sclerotherapy of Venous Malformations—Proof of Concept Study for Prediction of Clinical Outcome
Source: Diagnostics (Basel). 2022 Jun 9;12(6):1430. doi: 10.3390/diagnostics12061430 (PMC9222207; doi:10.3390/diagnostics12061430)
Supplement: Supplementary file 1 [file diagnostics-12-01430-s001.zip › Supplementary Material_diagnostics.pptx]

## Slide 1
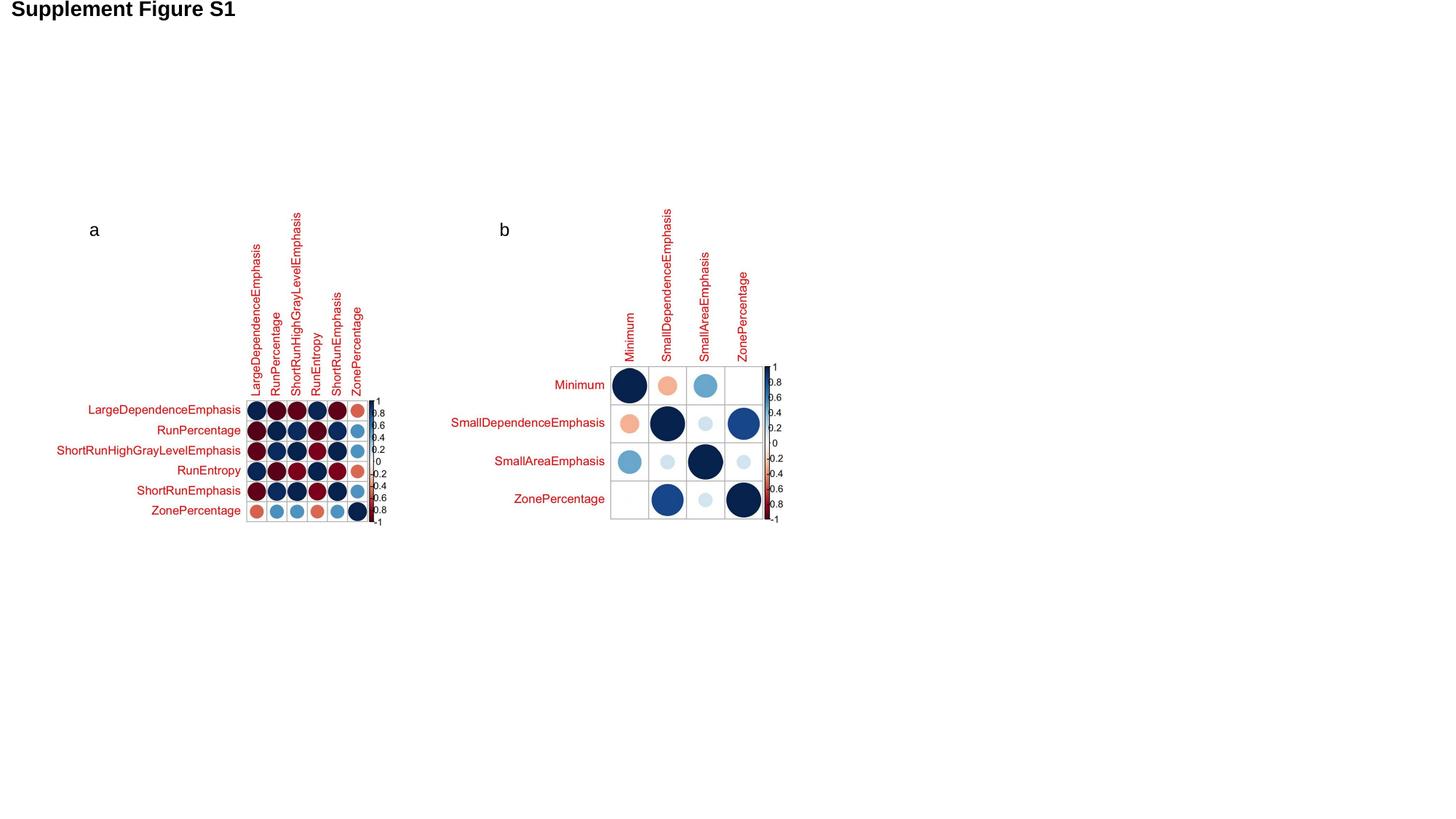

Supplement Figure S1
b
a

## Slide 2
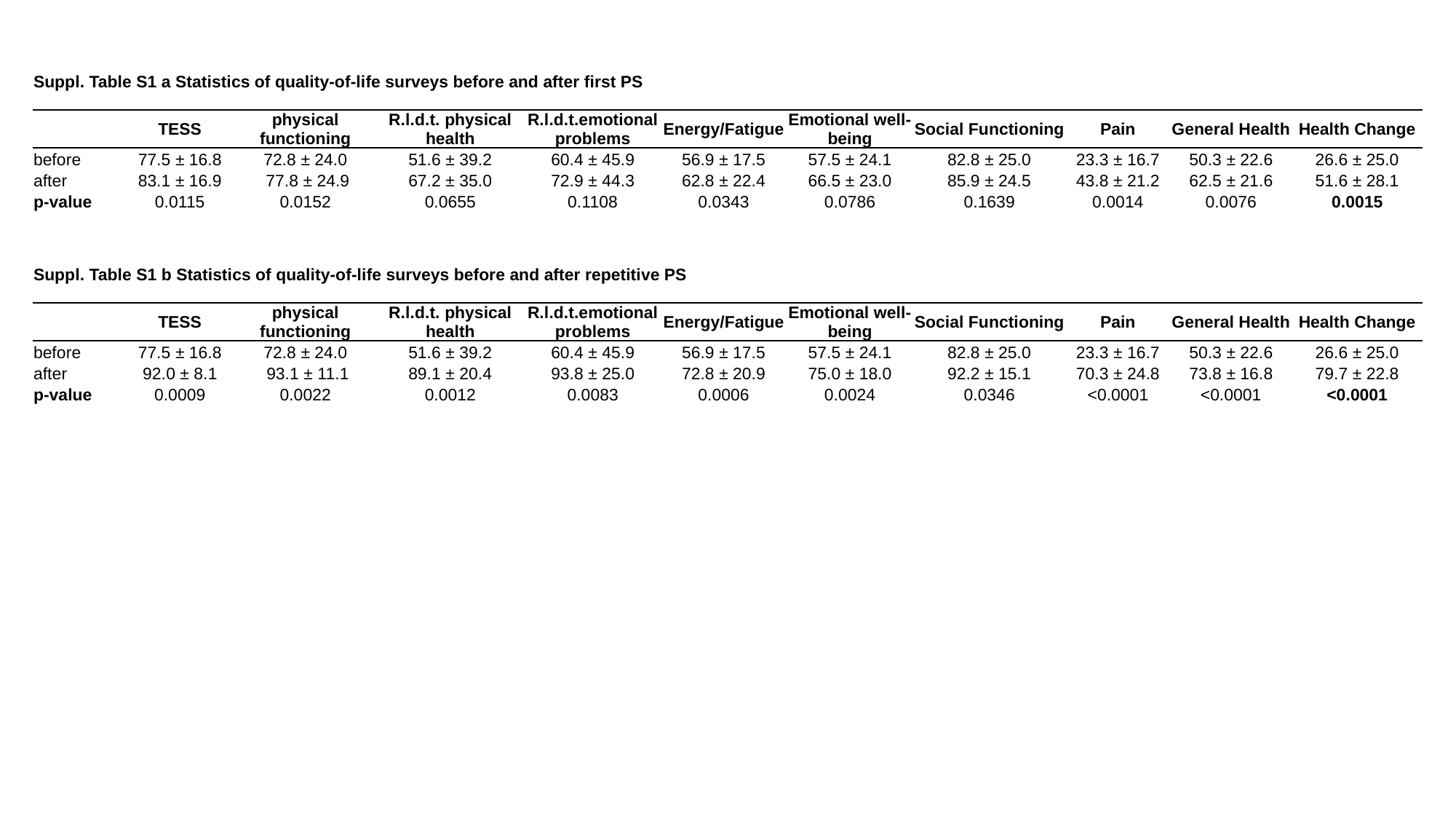

| Suppl. Table S1 a Statistics of quality-of-life surveys before and after first PS | | | | | | | | | | |
| --- | --- | --- | --- | --- | --- | --- | --- | --- | --- | --- |
| | TESS | physical functioning | R.l.d.t. physical health | R.l.d.t.emotional problems | Energy/Fatigue | Emotional well-being | Social Functioning | Pain | General Health | Health Change |
| before | 77.5 ± 16.8 | 72.8 ± 24.0 | 51.6 ± 39.2 | 60.4 ± 45.9 | 56.9 ± 17.5 | 57.5 ± 24.1 | 82.8 ± 25.0 | 23.3 ± 16.7 | 50.3 ± 22.6 | 26.6 ± 25.0 |
| after | 83.1 ± 16.9 | 77.8 ± 24.9 | 67.2 ± 35.0 | 72.9 ± 44.3 | 62.8 ± 22.4 | 66.5 ± 23.0 | 85.9 ± 24.5 | 43.8 ± 21.2 | 62.5 ± 21.6 | 51.6 ± 28.1 |
| p-value | 0.0115 | 0.0152 | 0.0655 | 0.1108 | 0.0343 | 0.0786 | 0.1639 | 0.0014 | 0.0076 | 0.0015 |
| Suppl. Table S1 b Statistics of quality-of-life surveys before and after repetitive PS | | | | | | | | | | |
| --- | --- | --- | --- | --- | --- | --- | --- | --- | --- | --- |
| | TESS | physical functioning | R.l.d.t. physical health | R.l.d.t.emotional problems | Energy/Fatigue | Emotional well-being | Social Functioning | Pain | General Health | Health Change |
| before | 77.5 ± 16.8 | 72.8 ± 24.0 | 51.6 ± 39.2 | 60.4 ± 45.9 | 56.9 ± 17.5 | 57.5 ± 24.1 | 82.8 ± 25.0 | 23.3 ± 16.7 | 50.3 ± 22.6 | 26.6 ± 25.0 |
| after | 92.0 ± 8.1 | 93.1 ± 11.1 | 89.1 ± 20.4 | 93.8 ± 25.0 | 72.8 ± 20.9 | 75.0 ± 18.0 | 92.2 ± 15.1 | 70.3 ± 24.8 | 73.8 ± 16.8 | 79.7 ± 22.8 |
| p-value | 0.0009 | 0.0022 | 0.0012 | 0.0083 | 0.0006 | 0.0024 | 0.0346 | <0.0001 | <0.0001 | <0.0001 |

## Slide 3
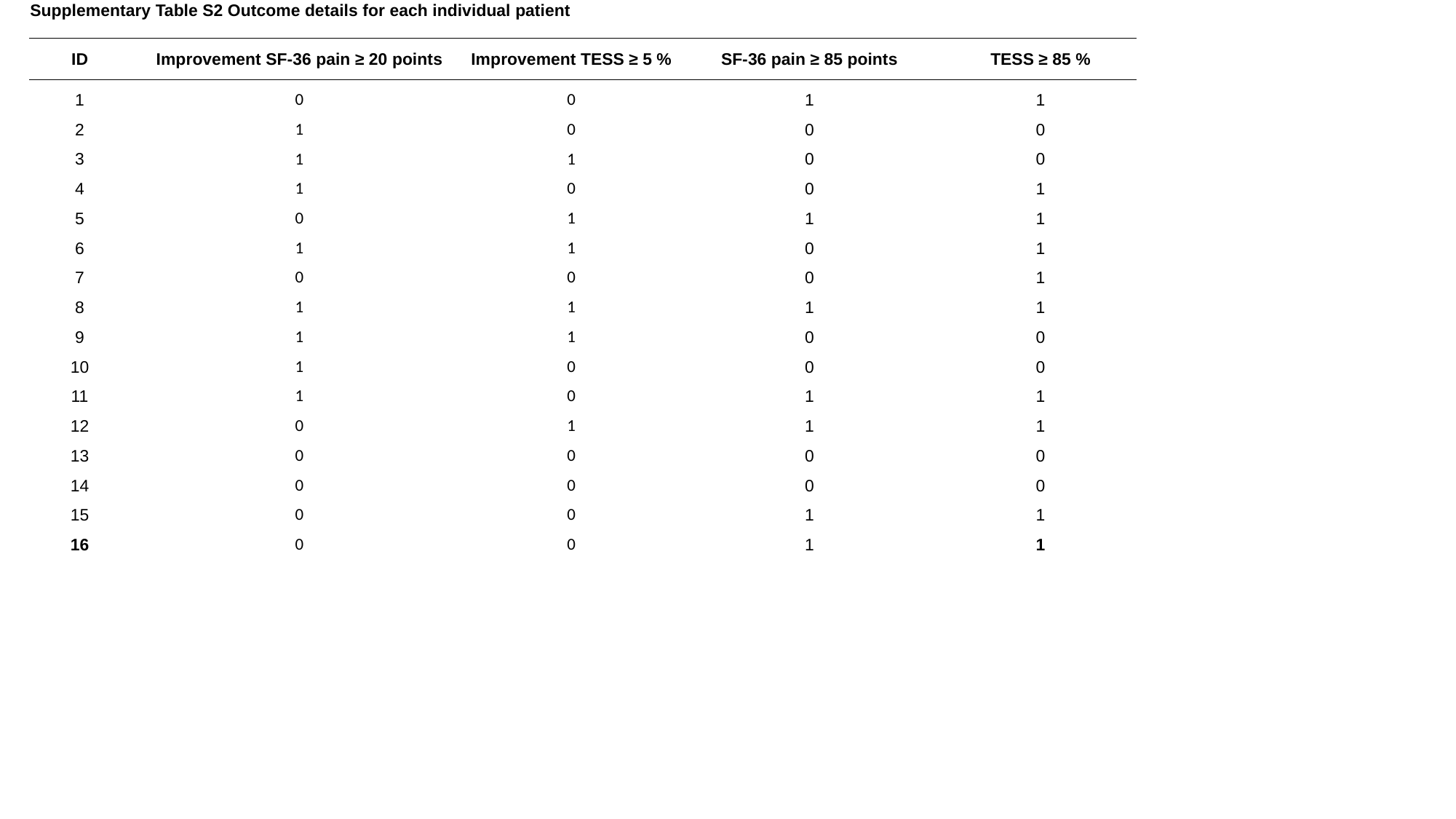

| Supplementary Table S2 Outcome details for each individual patient | | | | |
| --- | --- | --- | --- | --- |
| ID | Improvement SF-36 pain ≥ 20 points | Improvement TESS ≥ 5 % | SF-36 pain ≥ 85 points | TESS ≥ 85 % |
| 1 | 0 | 0 | 1 | 1 |
| 2 | 1 | 0 | 0 | 0 |
| 3 | 1 | 1 | 0 | 0 |
| 4 | 1 | 0 | 0 | 1 |
| 5 | 0 | 1 | 1 | 1 |
| 6 | 1 | 1 | 0 | 1 |
| 7 | 0 | 0 | 0 | 1 |
| 8 | 1 | 1 | 1 | 1 |
| 9 | 1 | 1 | 0 | 0 |
| 10 | 1 | 0 | 0 | 0 |
| 11 | 1 | 0 | 1 | 1 |
| 12 | 0 | 1 | 1 | 1 |
| 13 | 0 | 0 | 0 | 0 |
| 14 | 0 | 0 | 0 | 0 |
| 15 | 0 | 0 | 1 | 1 |
| 16 | 0 | 0 | 1 | 1 |

## Slide 4
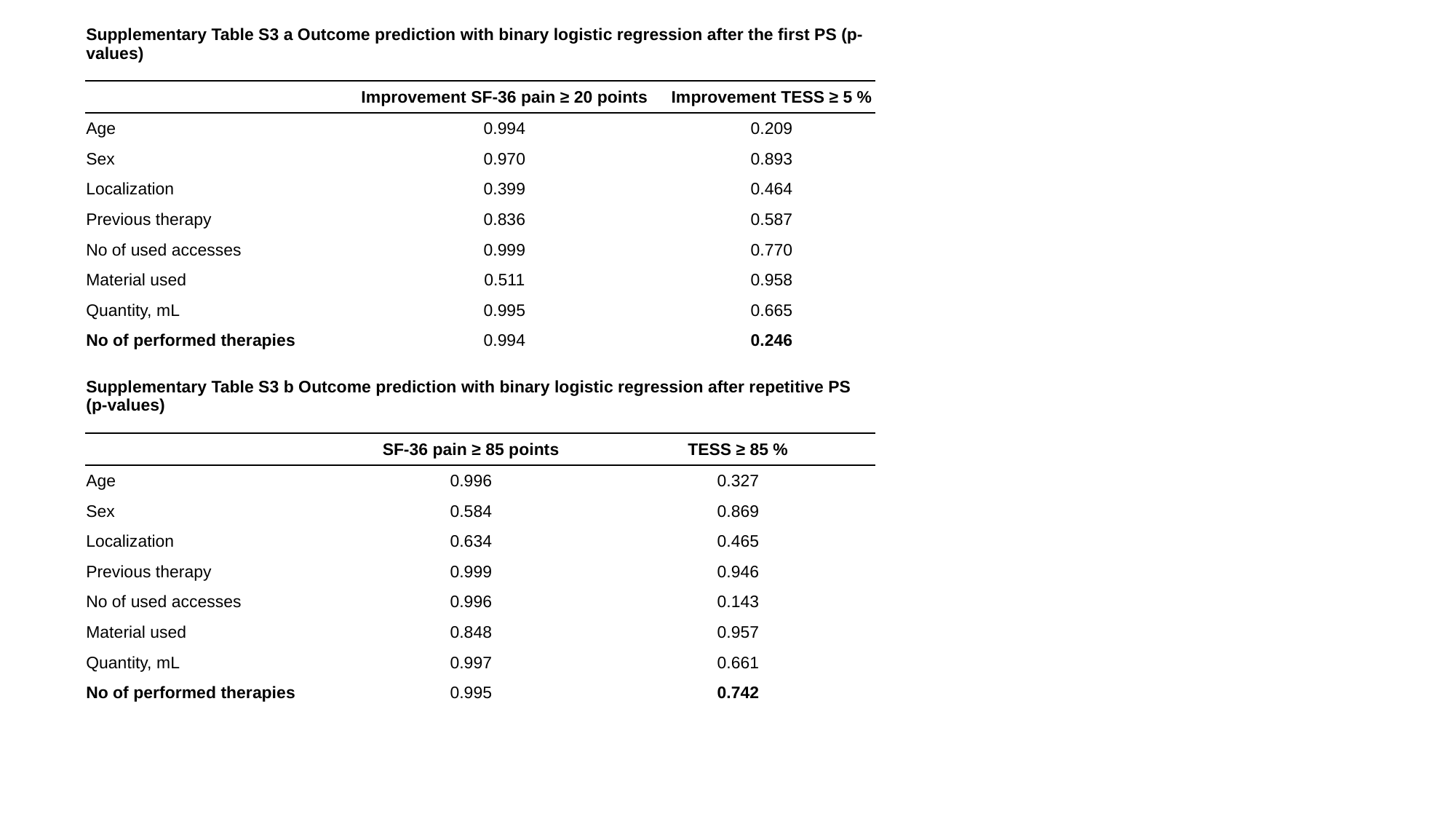

| Supplementary Table S3 a Outcome prediction with binary logistic regression after the first PS (p-values) | | |
| --- | --- | --- |
| | Improvement SF-36 pain ≥ 20 points | Improvement TESS ≥ 5 % |
| Age | 0.994 | 0.209 |
| Sex | 0.970 | 0.893 |
| Localization | 0.399 | 0.464 |
| Previous therapy | 0.836 | 0.587 |
| No of used accesses | 0.999 | 0.770 |
| Material used | 0.511 | 0.958 |
| Quantity, mL | 0.995 | 0.665 |
| No of performed therapies | 0.994 | 0.246 |
| Supplementary Table S3 b Outcome prediction with binary logistic regression after repetitive PS (p-values) | | |
| --- | --- | --- |
| | SF-36 pain ≥ 85 points | TESS ≥ 85 % |
| Age | 0.996 | 0.327 |
| Sex | 0.584 | 0.869 |
| Localization | 0.634 | 0.465 |
| Previous therapy | 0.999 | 0.946 |
| No of used accesses | 0.996 | 0.143 |
| Material used | 0.848 | 0.957 |
| Quantity, mL | 0.997 | 0.661 |
| No of performed therapies | 0.995 | 0.742 |
